# Effect of synthesized sulfonate derivatives as nucleating agents on crystallization behavior of poly(lactic acid)

**DOI:** 10.1080/15685551.2022.2072697

**Published:** 2022-05-06

**Authors:** Pasawat Jongpanya-Ngam, Rattikarn Khankrua, Manus Seadan, Supakij Suttiruengwong

**Affiliations:** a Department of Materials Science and Engineering, Faculty of Engineering and Industrial Technology, Silpakorn University, Nakhon Pathom, Thailand; b Department of Materials Engineering, Faculty of Engineering, Rajamangala University of Technology Rattanakosin, Nakhon Pathom, Thailand; c Department of Physics, Faculty of Science, Silpakorn University, Nakhon Pathom, Thailand

**Keywords:** Poly (lactic acid), crystallization, nucleating agent, aromatic sulfonate, transparency

## Abstract

The improvement of the crystallization of poly(lactic acid) (PLA) is one of the key areas to allow PLA to perform better at higher temperature and load bearing. Due to its slow crystallization rate, either organic or inorganic nucleating agents (NAs) can be used to improve the crystallization rate of PLA. In the case of organic NAs, aromatic sulfonate salt and bisamide compounds are promising ones because they can control better clarity. The aim of this work was to study the crystallization behavior of PLA using as-synthesized dimethyl 5-sulfoisophthalate sodium salt (SSIPA) as a nucleating agent in comparison with the commercial sulfonate salt (LAK-301). Two grades of PLA (PLA L105 and PLA 3251D) were used in this study. PLA samples were prepared by internal mixer and compression molding. All samples were investigated by DSC and POM. The results from DSC showed that after introducing the nucleating agents into PLA, the crystallinity in all samples was improved. The highest crystallinity at 57.48% was obtained from PLA L105/SSIPA1.0. Isothermal crystallization kinetics showed the improvement in overall crystallization rate of PLA with nucleating agents. The lowest half time crystallization obtained was 1.19 min for PLA L105/SSIPA1.0 at 135 °C. The results from POM indicated the substantial increase of the nucleus density and smaller spherulite size upon adding nucleating agents.

## 1. Introduction

Poly(lactic acid) (PLA) is one of the most widely used and the highest production capacity bioplastics in 2020 among those biodegradable plastics [1]. PLA is a renewable resource-based polymer that possesses good mechanical properties, processability, transparency, and biocompatibility [2]. Using PLA in the required compostable products is a good fit for the circular economy model, where the end-of-life can provide an organic recycling. Although PLA is now a major share in the compostable plastic category, it is not cheap and still needs to be improved in terms of working temperature and heat resistance. One of the major drawbacks of PLA is the slow crystallization rate which was very slow compared to commercial polymer; hence, PLA was almost amorphous after the process [3].

In order to solve this problem, there are some existing strategies to increase the crystallinity of PLA. One of them is the use of nucleating agents (NAs), which provide the nucleus site for the polymer, thus increasing nucleus density and crystallization rate of polymer [4]. Although the mechanism of each nucleating agent on polymers are different and not well understood, it is believed that it was induced from the molecular interaction between polymer and the surface of nucleating agents [3] and several researches reported the increase of crystallization rate with the use of different nucleating agents. Many kinds of nucleating agents have been reported in literatures for both organic and inorganic nucleating agents talc [5], hydrazide compound [6], calcium carbonate (CaCO_3_) [7], bisamide [8,9], sulfonate derivative [4,10,11], etc. Our research group has studied various organic nucleating agents based on the unsaturated bisamide compounds [8,9]. Khwanpipat T. et al. [8] synthesized the unsaturated N’N Ethylene bis(10-undecenamide) (EBU) together with peroxide as a nucleating agent for PLA. With 0.5 wt% of EBU, the half time crystallization of PLA reduces from 4.60 to 1.96 min at 110 °C. Apart from the bisamide compounds, other potential compounds reported as a nucleating agent for PLA was the sulfonate derivatives. Nagarajan, V. and his group [4] studied the effect of LAK-301, the commercial sulfonate derivative, as nucleating agent for PLA found that with 0.75 wt% half time crystallization of PLA reduces from 60.8 to 1.8 min at 140 °C. Many researches [4,10,11] have reported that successful increase of crystallization rate of PLA with sulfonate derivatives as a nucleating agent.

In this work, it was aimed to synthesize the sulfonate derivatives according to Oster, T. and his colleagues [12] and investigate the crystallization behavior of as-synthesized compounds. The chemical structure was similar to that used commercially except the cation of sulfonate salts. Two grades of PLA were selected to evaluate the nucleating agent performance. As-synthesized sodium dimethyl 5-sulfoisophthalate (SSIPA) was compared to potassium dimethyl 5-sulfoisophthalate, in terms of the crystallization kinetics and the spherulite sizes and nucleus density of PLA.

## 2. Materials and methods

### 2.1. Materials

PLA Luminy L105, injection grade with D-lactide content lower than 1% (melt flow index (MFI): 30 g/10 min (2.16 kg 190 °C) were kindly supplied by Total Corbion PLA Co. Ltd., Thailand. PLA Ingeo 3251D, injection grade with 1.4% D-lactide content (MFI: 35 g/10 min (2.16 kg 190°C) was purchased from NatureWorks LLC, USA. Potassium dimethyl 5-sulfoisophthalate (also known as LAK-301) from Takemoto Oil & Fat Co., Japan was used as received. 5-sulfoisophthalic acid (HSIPA) was purchased from Wako Pure Chemical Industries Ltd. Methanol, Glacial acetic acid and Sodium hydroxide was obtained from RCI Labscan Ltd., Thailand.

### 2.2. Synthesis of sodium dimethyl 5-sulfoisophthalate (SSIPA)

**Scheme 1.**
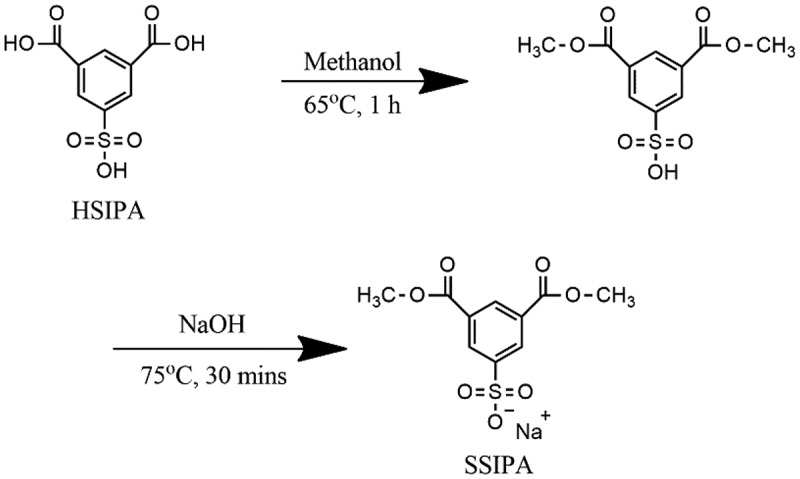
Synthesis of SSIPA.

Oster, T. and his co-workers were invented various method to synthesize metal salts of dialkyl ester of 5-sulfoisophthalic acid through esterification reaction and reaction with salts. One of them was sodium dimethyl 5-sulfoisophthalate [12]. As shown in Scheme 1, 27.6 g of 5-sulfoisophthalic acid (HSIPA) and 50 g of methanol were added into 250 ml three-necked round bottom flask at ambient temperature and then reflux at 65 °C for 1 hour. In another three-necked round bottom flask, 60 g of DI water, 6.6 g of glacial acetic acid and 8.2 g of sodium hydroxide (50% aqueous) were added and stirred at 70 °C for 1 hour. After 1 hour, cool the first flask down to 25–30 °C and then drop into latter flask over 10 minutes. The product slurry war stirred at 75 °C for 30 minutes then cool down to 25 °C, product then filter with cold DI water (0–5 °C) then dried in vacuum overnight to obtained white fine powder product that is the sodium dimethyl 5-sulfoisophthalate (SSIPA) (16.67 g, yield 60.3%).

### 2.3. Sample preparation

PLA and nucleating agents were first dried at 60 °C for 24 h and then blended in an internal mixer (MX105-D40L50, Charoentut Model, Charoentut, Co. Ltd., Samutprakarn, Thailand) at 190°C and 60 rpm for 10 min. After mixing, grounded PLA samples were compressed at 190 °C into thin film for polarized optical microscope (POM) study with thickness of around 100 μm, with 4 min preheat time and 1 min compression time. Table 1 showed the contents of nucleating agents added to PLA.

**Table 1.** Composition of PLA sample.

Sample	Content of substance (%)			
	PLA L105	PLA 3251D	LAK-301	SSIPA
L105	100.0	-	-	-
L105/LAK0.5	99.5	-	0.5	-
L105/LAK1.0	99.0	-	1.0	-
L105/SSIPA0.5	99.5	-	-	0.5
L105/SSIPA1.0	99.0	-	-	1.0
3251D	-	100.0	-	-
3251D/LAK0.5	-	99.5	0.5	-
3251D/LAK1.0	-	99.0	1.0	-
3251D/SSIPA0.5	-	99.5	-	0.5
3251D/SSIPA1.0	-	99.0	-	1.0

### 2.4. Characterization

#### 2.4.1. Differential scanning calorimetry (DSC)

DSC measurements were carried out using METTLER TOLEDO, DSC1 STAR instrument under a nitrogen atmosphere with the sample weight in range of 6–10 mg. Non-isothermal DSC was performed under heat-cool-heat cycle with temperature range of 30 to 200 °C and heating/cooling rate of 5 °C/min. For an isothermal study, the sample was first heated to 200 °C with heating of 15 °C/min and hold for 5 min to guarantee a total amorphous state, then sample was rapidly cooled with cooling rate 40 °C/min and hold at the desired temperature for 20 min. For neat PLA at high temperature were held for 3 h.

#### 2.4.2. Polarized optical microscope (POM)

POM was performed to study spherulite morphology by polarized optical microscope OPTIKA B-600 MET equipped with digital camera and Linkam THMS-600 hot stage. The sample was heated to 200 °C with heating rate of 15 °C/min and held for 10 min before being quickly cooled to 130 °C for neat PLA and 140 °C for PLA with nucleating agents to study the isothermal crystallization.

#### 2.4.3. X-ray diffraction (WAXD)

XRD samples were prepared by compression molding into disc samples with diameter of 1 inch and thickness of 2 mm. The samples were analyzed with X-ray diffractometer (LabX XRD-6100, Shimadzu, Bare Scientific Co. Ltd., Bangkok, Thailand) using Cu Kα radiation source (30 kV, 20 mA) with the 2θ angle range of 2–50° and the scanning rate of 12°/min.

## 3. Results and discussion

### 3.1. Synthesis and characterization of SSIPA

In this work, an organic nucleating agent was prepared according to T. Oster et al. method [12]. 5-sulfoisophthalic acid (HSIPA) and methanol were reacted through esterification reaction under 65°C for 60 min. The carboxylic group of HSIPA reacted with methanol and obtained 1,3-dimethyl 5-sulfoisophthalate. Then, sodium hydroxide was added and reacted with this intermediate under 75°C for 30 min, and finally obtained sodium dimethyl 5-sulfoisophthalate (SSIPA) as shown in Scheme 1.

**Figure 1.**
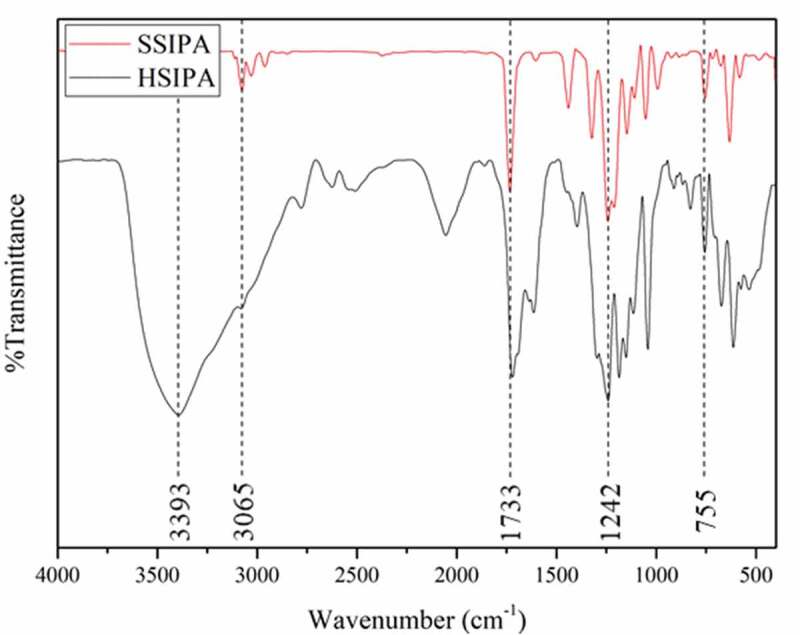
FTIR spectra of HSIPA and SSIPA.

In order to confirm the chemical reaction after synthesized, FTIR, ^1^H-NMR and EDS techniques were performed. Figure 1 illustrated the FTIR spectra of HSIPA and SSIPA. HSIPA showed characteristic peak around 3393 cm^−1^ that belonged to O-H stretching of carboxylic group in its structure Meanwhile, SSIPA showed peaked 3065 cm^−1^, which was assigned to C-H stretching from ester after esterification with methanol. Both spectra showed the peaks at around 1733, 1242 and 755 cm^−1^, which belonged to C = O stretching, S = O stretching and C-H aromatic, respectively, which existed in both substances.

**Figure 2.**
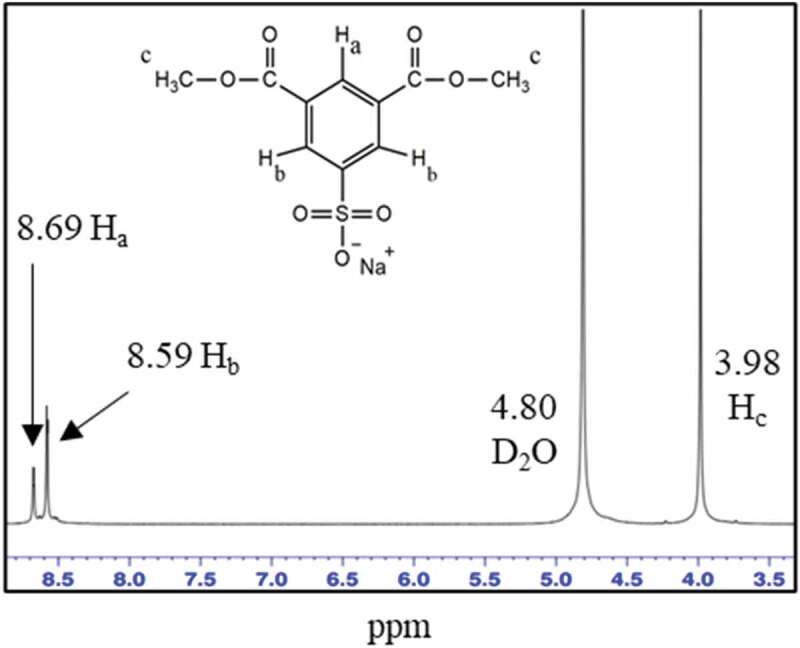
^1^H NMR spectrum of SSIPA.

Figure 2 showed ^1^H NMR spectrum of SSIPA with the signals at 8.69 and 8.59 ppm, which were attributed to the aromatic proton H_a_ and H_b_ in benzene ring, respectively. Signal at 3.98 ppm belonged to ester proton (-OCH_3_). The integral ratio of the signals at 8.69, 8.59, and 3.98 ppm were 1:2:6, which was in good agreement with SSIPA structure.

**Figure 3.**
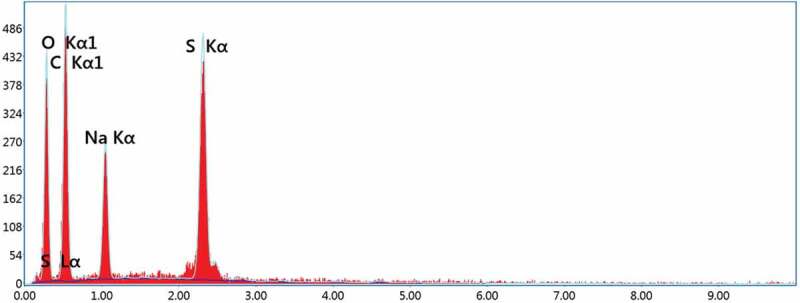
EDS spectrum of SSIPA.

Energy Dispersive X-Ray Spectroscopy (EDS) was also performed to confirm the successful reaction of sodium and the atomic ratio of synthesized substance as depicted in Figure 3. Table 2 showed the element concentration. The ratio between C, O, Na, and S atom was found to be about 10:7:1:1, which clearly confirmed the number of atoms in SSIPA structure. From these results, it confirmed the chemical reactions that were discussed before as well as demonstrated the successful synthesis of SSIPA from the reaction of HSIPA, methanol, and sodium hydroxide.

**Table 2.** Element concentration of SSIPA from EDS.

Element	Atomic (%)
C	52.80
O	35.08
Na	5.80
S	6.32

### 3.2. Non-isothermal DSC

**Figure 4.**
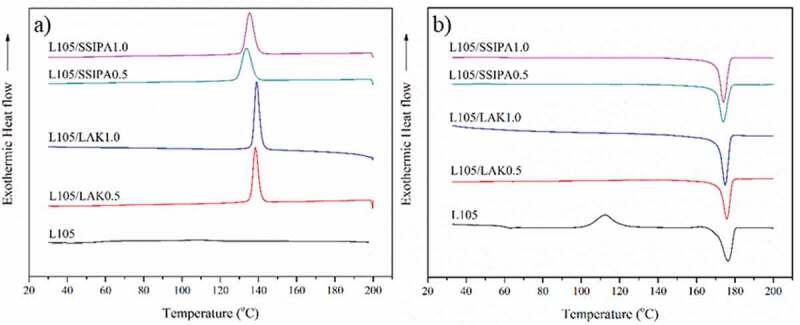
DSC thermograms of PLA L105 with and without nucleating agents on a) cooling and b) second heating cycle.

**Figure 5.**
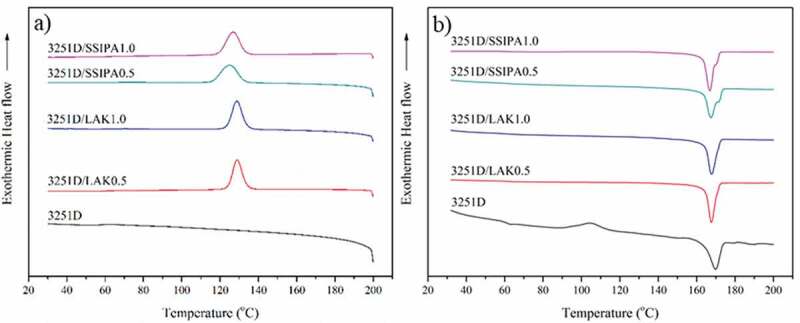
DSC thermograms of PLA 3251D with and without nucleating agents on a) cooling and b) second heating cycle.

The effect of nucleating agents on thermal characteristics of PLA was showed in Table 3. PLA is one of the compostable polymers known for its slow crystallization rate and low crystallinity [3]. Figure 4(a) and Figure 5(a) illustrated DSC thermogram at cooling cycle of PLA grade L105 and 3251D respectively. As expected, the crystallization peak did not observe during the cooling cycle in both PLA grades. This indicated that, in neat PLA, polymer chains were unable to rearrange and crystallize below the melt temperature, indicating that PLA had the slow crystallization rate. The crystallization peak in cooling cycle of both PLA grades appeared when applying with nucleating agents. This result indicated the improved crystallization of PLA during cooling cycle after obtained nucleating agent which provided nucleation site during crystallization periods. Figure 4(b) and Figure 5(b) show DSC thermogram at second heating cycle of PLA grade L105 and 3251D. The cold crystallization peak disappeared after introducing nucleating agents into both PLA grade. These phenomena supported that LAK and SSIPA acted as a potential nucleating agent for both PLA grades.

**Table 3.** Thermal characteristics of PLA and with nucleating agents.

Sample	Cooling	Second heating				
	T_c_ (°C)	T_cc_ (°C)	ΔH_cc_ (J/g)	T_m_ (°C)	ΔH_m_ (J/g)	%X_c_
Neat PLA L105	-	111.48	33.16	175.21	48.79	16.68
PLA L105/LAK0.5%	138.75	-	-	177.03	51.08	54.51
PLA L105/LAK1.0%	139.38	-	-	173.81	58.34	62.26
PLA L105/SSIPA0.5%	134.01	-	-	172.78	52.15	55.66
PLA L105/SSIPA1.0%	135.78	-	-	172.84	53.86	57.48
Neat PLA 3251D	-	104.50	23.76	169.48	43.23	20.78
PLA 3251D/LAK0.5%	129.14	-	-	167.34	46.91	50.06
PLA 3251D/LAK1.0%	128.95	-	-	167.40	49.76	53.11
PLA 3251D/SSIPA0.5%	126.05	-	-	167.10	45.56	48.62
PLA 3251D/SSIPA1.0%	126.99	-	-	166.65	49.97	53.33

In general, the addition of the nucleating agents could decrease the nucleation energy barrier and initiate the crystallization at higher temperatures when cooling. When compared two types of PLA, PLA L105 with similar nucleating agent types and contents showed higher crystallization temperature. The increase in the crystallization temperature might be from many different reasons. This may due to the effect of different D-lactide content in these PLA types. Since PLA L105 (less than 1%) possessed lower D-lactide content than PLA 3251D (1.4%). The addition of PDLA was also another reason. Shi, X. and his co-workers [13] studied the effect of stereo complex on crystallization behavior of PLA. After adding PDLA to PLLA, the crystallization temperature of non-isothermal DSC increased. However, in this study, no PDLA was added. If the stereo complex were to form, it would be observed at the temperature above 210 °C [8,13]. In term of nucleating agent types, addition of LAK showed slightly higher crystallization temperature when compared with SSIPA in both PLA types. This should be due to the effect of the difference in the cation salt at the sulfonate group among these nucleating agents. In addition, the difference nucleating agent content slightly affected the crystallization temperature of PLA. PLA L105 with nucleating agent tended to increase with increasing nucleating agent contents in both LAK and SSIPA. However, this phenomenon did not occur in the case of PLA 3251D.

(1) $$\%X_{c}=\;\frac{\Delta H_{m}-i\Delta H_{cc}}{\omega_{PLA}{\Delta H}_{m}^{0}}$$

where *ΔH_m_* is the enthalpy of melting (J/g), *ΔH_cc_* is the enthalpy of cold crystallization (J/g), *ω_PLA_* is the weight fraction of PLA and *ΔH^0^_m_* is the enthalpy of total crystalline melting of PLA (93.7 J/g) [4].

The percentage crystallinity (*%X_c_*), was calculated from equation (1). Since the selected PLA L105 and 3251D had high stereochemical purity (≥ 99% L-isomer), it was expected to have high crystallinity to start with. Both were used for injection molding. The increase crystallinity of both grades would ease the processing time and final properties of the production. From Table 3, it could be seen that both PLA grades possessed the percentage crystallinity of 16.68% and 20.78% for neat PLA L105 and neat PLA 3251D respectively. In our study for all neat PLA grades, it should be noted that high cold crystallization enthalpy was observed and disappeared when nucleating agents were added. The addition of nucleating agent could promote the crystallization of PLA, resulting in an increased degree of crystallinity in all cases. The effect of different nucleating agent content on crystallinity of both PLA also observed in this work. *%X_c_* of PLA after applying with nucleating agent tended to increase with increasing nucleating agent contents. This similar results were also observed by Nagarajan et al. [4], who studied an organic aromatic sulfonate compound (LAK) as a nucleating agent for PLLA. They found that the addition of 0.25 wt% LAK increased the crystallinity of PLLA from 10% (neat PLLA) to 45%. Additionally, the crystallization mechanism for such compound was not well understood and required more studies.

### 3.3. Isothermal DSC

The isothermal crystallization behavior of PLA with nucleating agents was investigated at a range of temperature between 135–150 °C for PLA L105 and 125–140 °C for PLA 3251D, temperature was taken from the crystallization temperature of each PLA with nucleating agents. For neat PLA, the crystallization temperature in the range of 105–120 °C were suggested by S.C. Schmidt and his group [3]. Thus, the cold crystallization temperature was used in this study.

A simple way to study the isothermal crystallization was the evaluation of the relative crystallinity (*X_t_*) as a function of time at different crystallization temperatures. Relative crystallinity was expressed as the ratio of the area of the crystallization time to the total area of exotherm peak [4].

(2) $$X_{t}=\frac{\int_{0}^{t}(dH/dt)dt}{\int_{0}^{\infty }(dH/dt)dt}$$

Where *dH/dt* is the rate of heat flow during the crystallization process at time *t*.

**Figure 6.**
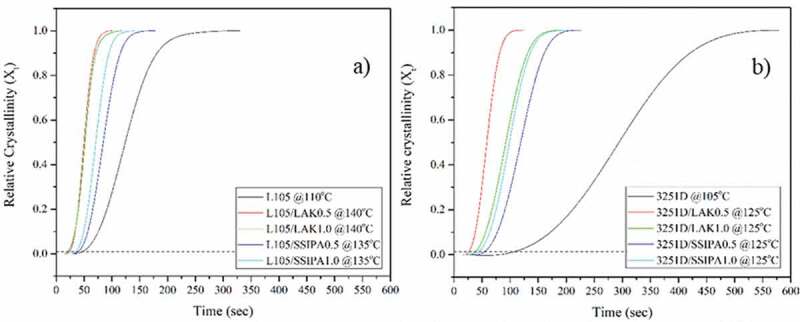
Relative crystallinity of PLA and PLA with nucleating agents a) L105 and b) 3251D.

Figure 6(a,b) showed the relative crystallinity of PLA, at the optimum investigated temperature, with time. At dash line, about 1–2% crystallinity or the nucleation step, it was seen that the induction time of nucleation step decreased after introducing nucleating agent into PLA. Also, from the curve, it was obvious that LAK had lower induction time than SSIPA. The induction time of PLA with LAK 0.5 wt% was higher than 1.0 wt% for PLA L105 but lower in the case of PLA 3251D.

For SSIPA, 1.0 wt% of nucleating agent exhibited lower induction time in both PLA. When comparing two types of PLA with same kind and content of nucleating agent, PLA L105 had slightly lower induction time than PLA 3251D. We can also observe the growth rate of crystallization from slope of the curve, which was found that the slope increased after introducing nucleating agent into PLA. This indicated that the growth rate of crystallization of PLA was increased. We can clearly observe that PLA with LAK have higher slope than SSIPA. The effect of content of nucleating agent exhibited differently; PLA with 0.5 wt% of LAK had higher slope than 1.0 wt% but PLA with 1.0 wt% of SSIPA showed higher slope than 0.5 wt%. Upon comparison between two types of PLA with same kind of nucleating agent, PLA L105 had higher slope than PLA 3251D.

Generally, Avrami theory was used to explain and analyze the isothermal crystallization kinetics parameters. Avrami method relates relative crystallinity and time by the equation (3), which can be rewritten into equation (4)

(3) $$1-X_{t}=exp\left(-kt^{n}\right)$$

(4) $$\ln\left[-\ln\left(1-X_{t}\right)\right]=nln\left(t\right)+ln\left(k\right)$$

Where *k* is the crystallization rate constant (include nucleation and growth rate) and *n* is the Avrami exponent, which depends on crystal growth dynamic, nucleation type and crystal geometry [14].

The double log plot showed the deviation from linearity on beginning and ending of crystallization caused by the initial and secondary crystallization, which occurred because of the completion of the crystal [15]. Thus, the value in the range of 20–80% of relative crystallinity was used to fit the equation to obtain n and k value.

From Tables 4 and 5, n values of neat PLA at lower temperature were in the range of 3.4–3.8, which indicated that crystals were mainly growth in three dimensions with heterogenous nucleation. At higher temperature, the temperatures were chosen from crystallization temperature peak of PLA with nucleating agent, n value in range of 1.4–1.8, which indicated that two-dimensional crystal growth with heterogenous nucleation was favored. After introducing nucleating agent, n value increased to 4. This indicated that crystals growth in three dimensions with homogenous nucleation are favored.

**Table 4.** Isothermal crystallization kinetics parameters of PLA L105.

Temperature	k (min^−n^)	n	t_1/2_ (min)
PLA L105			
110 °C	0.05	3.46	2.07
140 °C	0.00	1.44	60.70
PLA L105/LAK0.5			
135 °C	18.06	4.21	0.46
140 °C	1.47	4.35	0.84
145 °C	0.02	4.57	2.13
150 °C	0.00	4.78	5.96
PLA L105/LAK1.0			
135 °C	11.22	4.94	0.56
140 °C	1.28	4.14	0.86
145 °C	0.00	4.64	2.67
150 °C	0.00	4.68	7.36
PLA L105/SSIPA0.5			
135 °C	0.16	4.23	1.41
140 °C	0.01	4.62	2.36
145 °C	0.00	4.70	4.60
150 °C	0.00	4.32	10.46
PLA L105/SSIPA1.0			
135 °C	0.30	4.59	1.19
140 °C	0.02	4.73	2.03
145 °C	0.00	4.83	3.96
150 °C	0.00	4.48	8.01

**Table 5.** Isothermal crystallization kinetics parameters of PLA 3251D.

Temperature	k (min^−n^)	n	t_1/2_ (min)
PLA 3251D			
105 °C	0.00	3.37	4.90
130 °C	0.00	1.83	65.21
PLA 3251D/LAK0.5			
125 °C	0.74	3.90	0.98
130 °C	0.33	4.48	1.17
135 °C	0.04	4.64	1.78
140 °C	0.00	4.79	4.01
PLA 3251D/LAK1.0			
125 °C	0.14	3.72	1.54
130 °C	0.10	3.84	1.64
135 °C	0.01	4.23	2.32
140 °C	0.00	4.43	4.54
PLA 3251D/SSIPA0.5			
125 °C	0.04	4.06	1.98
130 °C	0.02	4.06	2.29
135 °C	0.00	4.34	3.34
140 °C	0.00	4.70	5.97
PLA 3251D/SSIPA1.0			
125 °C	0.09	3.95	1.65
130 °C	0.03	4.14	2.07
135 °C	0.00	4.40	2.97
140 °C	0.00	4.47	5.36

Crystallization kinetics was discussed based on half time crystallization (*t_1/2_*), time used to achieve 50% crystallinity, which was calculated by the equation (5)

(5) $$t_{1/2}=(\ln2/k{)}^{1/n}$$

The calculated *t_1/2_* was showed in Tables 4 and 5. For PLA with nucleating agents, the half time crystallization tended to increase with increasing crystallization temperature. PLA with LAK showed lower half time crystallization than SSIPA when compared with the same nucleating agent content and crystallization temperature. PLA L105 showed lower half time crystallization than PLA 3251D with same content and nucleating agent. This might due to the stereo complex that have been mentioned in previous result. Shi, X. and his colleagues [13] also obtained the similar results on isothermal crystallization behavior of PLA with stereo complex. From the investigated content of nucleating agent, PLA with 0.5 wt% of LAK showed lower half time crystallization than 1.0 wt%. But in case of SSIPA, PLA with 1.0 wt% of SSIPA showed lower half time crystallization. Lowest half time crystallization obtained from PLA with SSIPA was 1.19 min at 135 °C for PLA L105/SSIPA1.0 and 1.65 min at 125 °C for PLA 3251D/SSIPA1.0.

**Figure 7.**
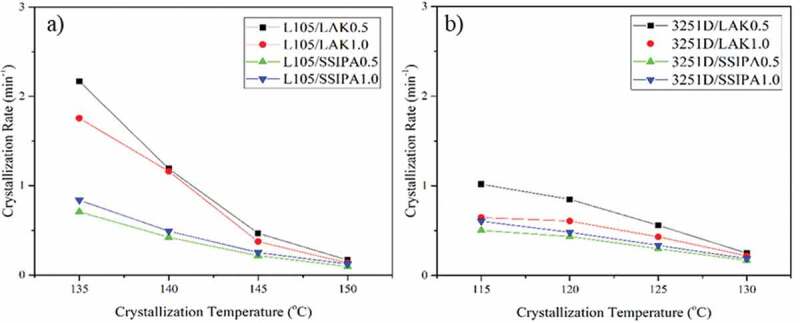
Crystallization rate of PLA and PLA with nucleating agents a) L105 and b) 3251D.

The crystallization rate was explained by 1/*t_1/2_*, because k values had in unit of min^−n^ and depended on n value, which also relied on the crystallization temperature. Thus, k values cannot directly compare. The plotted between 1/*t_1/2_* and crystallization temperature were showed in Figure 7. It was found that at higher crystallization temperature, the crystallization rate decreased in all samples. We can also observe that PLA with LAK 0.5 wt% showed higher crystallization rate than LAK 1.0 wt% at same crystallization temperature. The addition of 1.0 wt% of LAK might exceed the maximum contents for crystallization improvement, V. Nagarajan et al. [4] also observed that PLA with 0.75 wt% of LAK exhibited higher crystallization rate than 1.0 wt%. From Table 4 and Figure 7(a), it was found that PLA with 0.5 wt% and 1.0 wt% LAK at 135 °C had higher k value, crystallization rate and lower half time crystallization. At isothermal temperature of 135 °C, some crystals have been formed before reaching the investigated temperature from the isothermal crystallization peak. The similar findings were observed by V. Nagarajan and his group [4].

### 3.4. Polarized optical microscope

**Figure 8.**
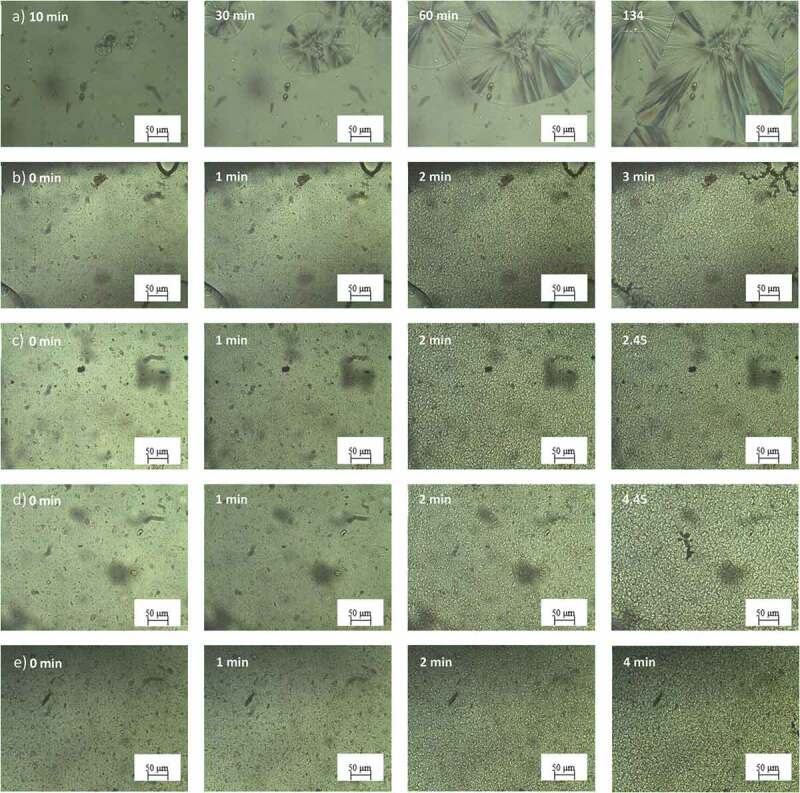
POM images of a) neat PLA L105 and PLA L105 with b) LAK 0.5 wt%, c) LAK 1.0 wt%, d) SSIPA 0.5 wt% and e) SSIPA 1.0 wt% at 130°C.

**Figure 9.**
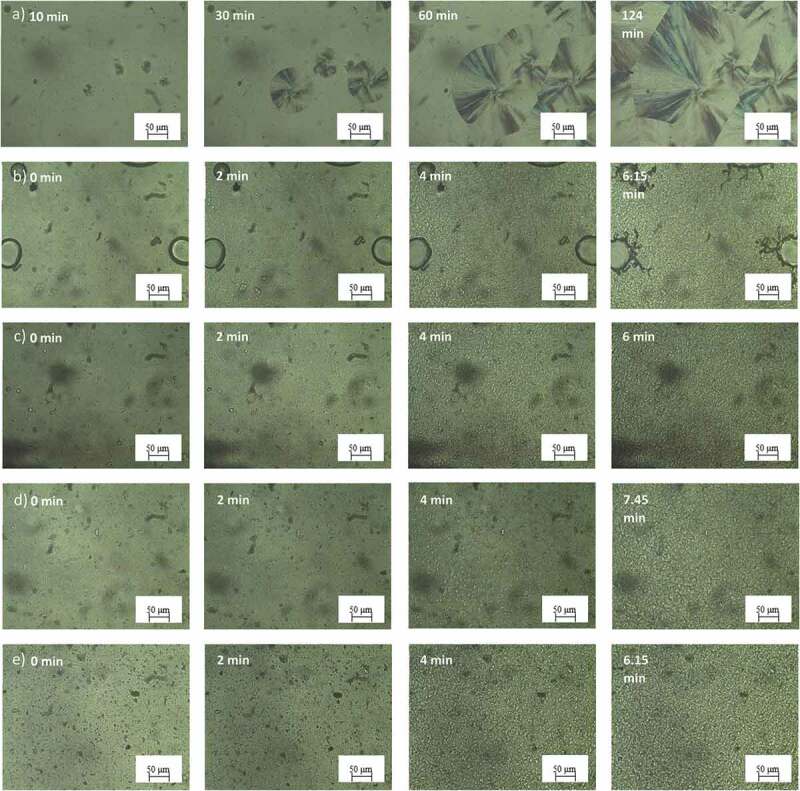
POM images of a) neat PLA 3251D and PLA 3251D with b) LAK 0.5 wt%, c) LAK 1.0 wt%, d) SSIPA 0.5 wt% and e) SSIPA 1.0 wt% at 130°C.

POM was performed to study the morphology of spherulites. Figure 8 and Figure 9 showed POM images of PLA with and without nucleating agents at different times. As expected, both PLA showed slow crystallization rate as observed by a slow spherulite growth during testing which used 134 and 124 minutes for full growth of PLA L105 and 3251D crystals, respectively as illustrated in Figure 8(a) and Figure 9(a). After introducing the nucleating agent into PLA, it was also clearly seen that the nucleus density was substantially increased whereas the spherulite sizes were dramatically decreased times for fully crystal growth in both nucleating agent system dramatically decreased. PLA with LAK show higher crystallinity compared with PLA with SSIPA at the simultaneous time indicates that PLA with LAK have higher crystallization rate which is agreeable with the isothermal DSC results.

**Figure 10.**
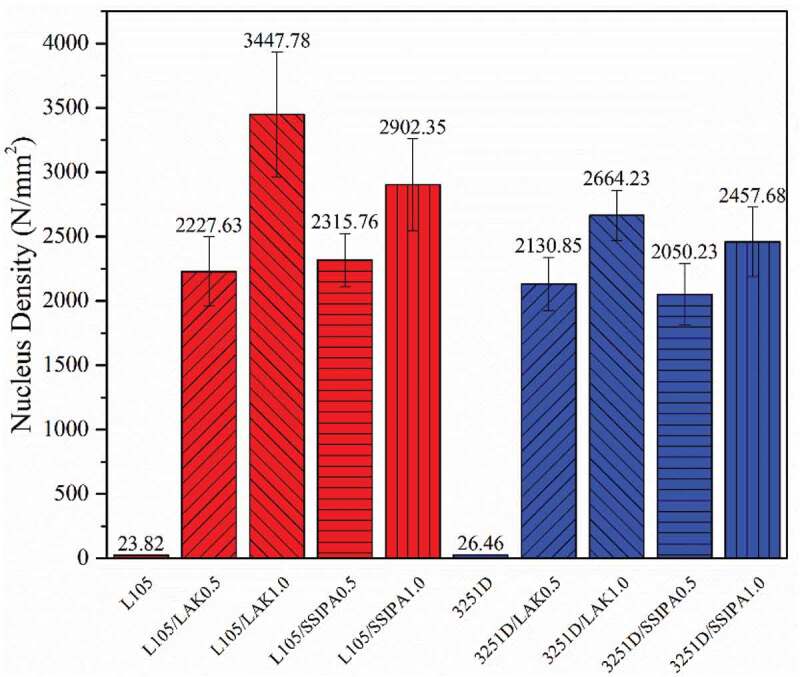
Nucleus density of PLA and PLA with nucleating agent.

The effect of nucleating agents on nucleus density of PLA was discussed. From Figure 10 illustrated nucleus density of PLA and PLA with nucleating agents, the PLA L105 and PLA 3251D showed low nucleus density at 23.82 and 26.46 N/mm^2^, respectively. When nucleating agents were applied, it was also clearly seen that the nucleus density was substantially increased in both SSIPA and LAK systems. In the SSIPA system, nucleus density of both PLA L105 and 3251D increased to 2902.35 and 2457.68 N/mm^2^ after applied with SSIPA at 1.0%wt. Meanwhile, the same tendency is also observed in the LAK nucleating agent system. Nucleus density of PLA L105 and 3251D increased to 2902.35 and 2457.68 N/mm^2^ after applied with LAK at 1.0%wt.

**Figure 11.**
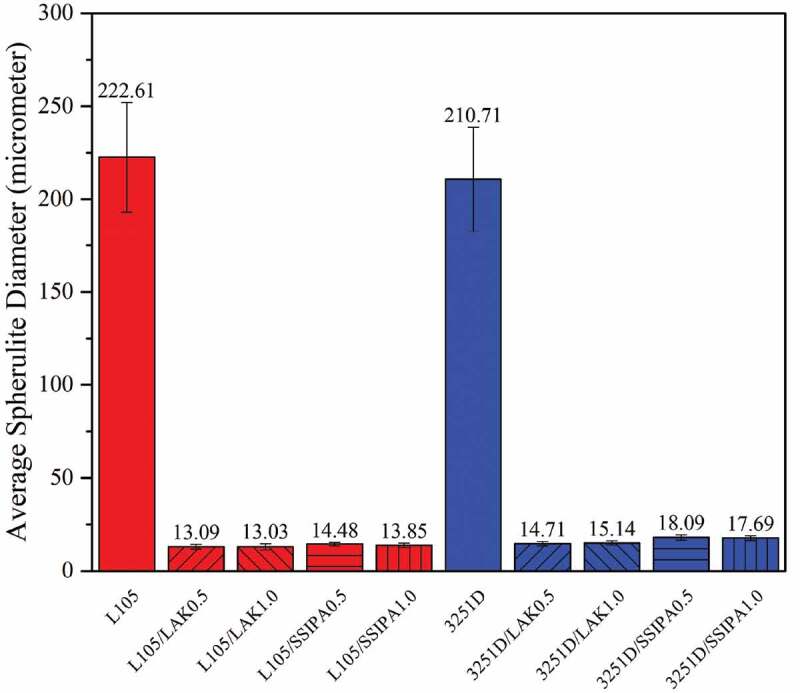
Average spherulite diameters of PLA and PLA with nucleating agent.

Furthermore, nucleating agents also affected spherulite size of both PLA L105 and 3251D. Figure 11 showed average spherulite diameter of PLA and PLA with nucleating agents. The spherulite sizes were dramatically decreased after applying a nucleating agent. Spherulite size of both PLA grades reduced approximately 15 times when compared with neat PLA. In addition, nucleating agent content also affect nucleus density of PLA, nucleus density of PLA with the same nucleating agent at 1.0 wt% was higher than that of 0.5 wt%, but the average spherulite sizes of PLA with 0.5 wt% and 1.0 wt% of the same nucleating agent remained very similar, as shown in Figure 11.

When comparing neat PLA and PLA with nucleating agents, nucleation and crystal growth of neat PLA were considerably slow. Nucleating agent was expected to provide nucleation site for PLA. PLA did not have to undergo the nucleation itself, thus the surface energy barrier for nucleation was reduced [15], and also increased the nucleus density. Considering the images from POM together with the effect of the nucleating agent mentioned above, LAK and SSIPA could serve as an effective nucleating agent for both PLA grades, but in in different potential molding temperatures. In terms of the chemical structures, both LAK and SSIPA were very similar and should provide the same crystallization mechanism.

### 3.5. X-ray diffraction analysis

**Figure 12.**
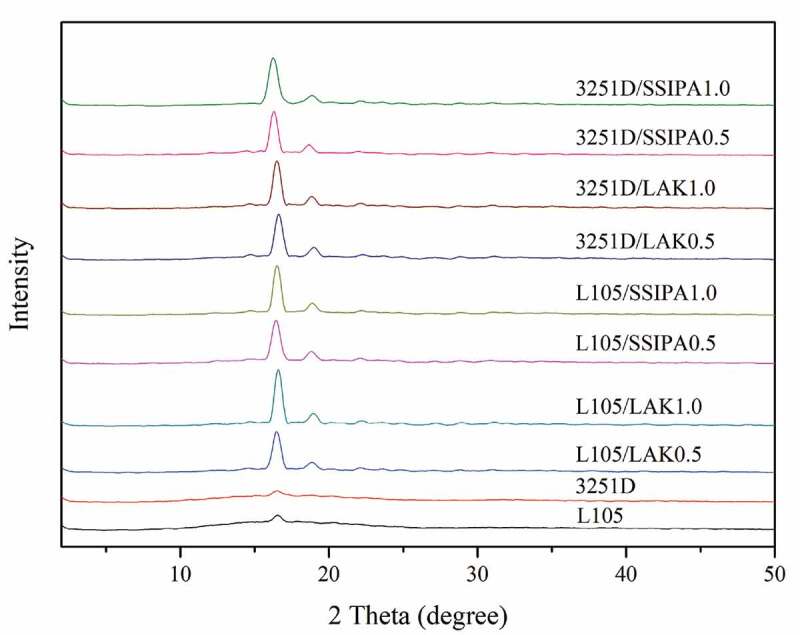
XRD patterns of neat PLA and PLA with nucleating agent.

XRD patterns of neat PLA and PLA with different nucleating agents were shown in Figure 12. As expected, XRD pattern of neat PLA showed a broad diffraction curve indicating an amorphous nature of PLA with a weak diffraction peak at 2θ equal to 16.50°, which indicated the semicrystalline behavior part of PLA. Introducing nucleating agent substantially increased the intensity of peak at 16.34–16.50°, which was indexed as (200/110), a characteristic peak of α crystal form, and new peak appeared at 18.9–19.12°, which was indexed as (203), a characteristic peak of α’ crystal form of PLA [16]. This result confirmed an enhanced the crystallization of PLA after applying SSIPA or LAK nucleating agents, which are in good agreement with DSC and POM results. However, there was no significantly shift of diffraction peak in both SSIPA and LAK samples, meaning that both nucleating agents did not affect the crystal form of PLA.

## 4. Conclusion

In this research, SSIPA was successfully synthesized. The crystallization behavior of PLA with SSIPA as a nucleating agent were investigated in comparison with LAK. The presence of SSIPA caused the cold crystallization peak of neat PLA to disappear and induced new crystallization peak. The highest crystallinity was achieved for PLA L105 and PLA 3251D with SSIPA was 57.48% and 53.33%, respectively. Overall crystallization rate from isothermal crystallization studies was much faster for PLA with nucleating agent than that of neat PLA. The fastest crystallization rate of PLA with SSIPA was obtained at 135 °C for PLA L105 and 125 °C for PLA 3251D, both with 1.0 wt% of SSIPA. The lowest half time crystallization obtained was found to be 1.19 min at 135 °C for PLA L105/SSIPA1.0. The n values of PLA with nucleating agent increased from around 3 to 4, implying that the nucleating agent changed the favored nucleation from heterogenous to homogenous nucleation with spherical three-dimensional growth. POM images showed the smaller size of spherulites and also higher nucleus density. WAXD results revealed the improve crystallinity after addition of nucleating agent and no effect of nucleating agent on crystal form was observed. From all the results, it could be summarized that the sulfonate derivative, SSIPA, could potentially be used as the effective nucleating agent for PLA, where the different molding temperatures and PLA grades played a significant role for the right processing conditions.
